# Valuable Serum Markers in Pulmonary Alveolar Proteinosis

**DOI:** 10.1155/2019/9709531

**Published:** 2019-11-11

**Authors:** Shenyun Shi, Lulu Chen, Xiaohua Qiu, Qi Zhao, Yonglong Xiao, Xin Yan

**Affiliations:** ^1^Department of Respiratory Medicine, Nanjing Drum Tower Hospital, Clinical College of Nanjing Medical University, Nanjing, 210008 Jiangsu, China; ^2^Department of Respiratory Medicine, Nanjing Drum Tower Hospital, Nanjing University Medical School, Nanjing 210008, China

## Abstract

**Objective:**

Several serum markers were reported to reflect the severity of pulmonary alveolar proteinosis (PAP). The aim of this study is to investigate a reliable and facile marker to access and monitor the clinical course of PAP in a large cohort.

**Methods:**

PAP patients from January 2010 to June 2018 were enrolled. Hospital records were used as data sources. The levels of various serum indicators were detected. We evaluated the correlation between pulmonary function test results and clinical variables.

**Results:**

Diffusion capacity for carbon monoxide (DLCO) level was positively correlated with the level of high-density lipoprotein cholesterol (HDL-C) (*P* < 0.05) in 122 patients of PAP at baseline. The levels of HDL-C and DLCO significantly increased while carcinoembryonic antigen (CEA), CYFRA21-1, neuron-specific enolase (NSE), and lactic dehydrogenase (LDH) levels decreased six months after granulocyte-macrophage colony-stimulating factor (GM-CSF) inhalation therapy between 14 patients with PAP. Nevertheless, the increased DLCO was significantly correlated with decreased CEA (*r* = ‐0.579, *P* = 0.031) and CYFRA 21-1 (*r* = ‐0.632, *P* = 0.015). In 10 PAP patients without GM-CSF inhalation therapy, HDL-C and DLCO significantly decreased while NSE and LDH levels increased after six months of follow-up. The decreased DLCO was significantly correlated with increased LDH (*r* = ‐0.694, *P* = 0.026).

**Conclusions:**

Serum CEA, CYFRA21-1, and LDH are valuable serum markers for the evaluation of disease activity of PAP and may predict the response to treatment of PAP.

## 1. Background

Pulmonary alveolar proteinosis (PAP) is a rare and severe disease in which characterized by the accumulation of lipoproteinaceous material within the alveoli and terminal airways [1]. The clinical feature of the disease is progressive exertional dyspnea of insidious onset and cough, also with a high risk of pulmonary infections causing respiratory insufficiency. PAP has been described in three forms clinically: primary PAP that can be autoimmune PAP which is associated with elevated levels of autoantibodies against granulocyte-macrophage colony-stimulating factor (GM-CSF) or hereditary PAP which is due to gene mutations of GM-CSF receptor alpha and beta genes (CSF2RA and CSF2RB, respectively), secondary PAP, and congenital PAP. More than 90% pulmonary alveolar proteinosis is autoimmune [2].

It has been reported that in patients with primary PAP, some pulmonary cytokine level could be used to assess and monitor disease progression. For example, the level of tumor markers, which is elevated both in the serum and bronchoalveolar lavage fluid (BALF) of humans with acquired pulmonary alveolar proteinosis [3]. Similarly, the level of serum lipids is also elevated in the serum of humans with the acquired disease [4]. However, these results have not yet been performed in larger PAP population samples. It is unknown whether these markers are changed with the course of PAP. This study is intended to find a reliable and facile marker to access and manage the disease activity of PAP.

## 2. Subjects and Methods

### 2.1. Subjects

This study included 122 PAP patients who were recruited from the inpatient of the Department of Respiration of Nanjing Drum Tower Hospital between January 2010 and June 2018 and diagnosed by transbronchial lung biopsy. All subjects gave informed written consent to participate. Patients were excluded from the study if they had sarcoidosis, occupational lung disease, idiopathic pulmonary fibrosis, pulmonary tuberculosis, COPD, and cancer. According to the disease severity score (DSS) of PAP defined by Inoue et al. [5], the grades were as follows: grade 1, PaO2 ≥ 70 mmHg without respiratory symptoms; grade 2, PaO2 ≥ 70 mmHg with respiratory symptoms; grade 3, 70 mmHg > PaO2 ≥ 60 mmHg; grade 4, 60 mmHg > PaO2 ≥ 50 mmHg; and grade 5, PaO2 < 50 mmHg. Arterial blood gas of PAP patients was analyzed when they were breathing room air. Among 122 PAP patients in this study, the disease severity score of 9 PAP patients at the baseline was classified into grade 1. The DSS score of 52 PAP patients was classified into grade 2. There were 30 PAP patients whose DSS score were classified into grade 3. There were 20 and 11 PAP patients who were, respectively, classified into grade 4 and grade 5.

### 2.2. Method

#### 2.2.1. Laboratory Tests

Routine biochemical analyses including plasma lipids and serum lactate dehydrogenase (LDH) were measured with commercial kits using an automated chemistry analyzer (Chemistry Analyzer Au2700, Olympus Medical Engineering Company, Japan). The white blood cell count (WBCC), neutrophil, lymphocyte, and monocyte differentials were determined using an automated blood cell counter (Beckman Coulter Ireland Inc. Mervue, Galway, Ireland). CEA, CYFRA 21-1, and neuron-specific enolase (NSE) were measured with an enzyme immunoradiometric assay kit (TFB, Tokyo, Japan). IgA, IgG, IgM, IgE, C3, and C4 were determined using immunoturbidimetry. Pulmonary function tests (Fukuda Sangyo, Japan) were performed in PAP patients.

#### 2.2.2. GM-CSF Inhalation

In our hospital, there are only a small number of patients treated with whole lung lavage (WLL). However, the follow-up data of the patients treated with WLL is not available. So far, there have been no patients treated with rituximab. In this study, fourteen patients were treated with GM-CSF inhalation. These patients elected to initiate inhaled GM-CSF (200 mg) twice daily, three times per week, administered two weeks per month for six months.

#### 2.2.3. Statistical Analysis

Data are expressed as mean ± SD. Correlations between DLCO-SB and other parameters were evaluated by Pearson**'s** correlation analysis. The differences of DLCO-SB and other parameters before and after GM-CSF inhalation or between baseline and six-month follow-up without GM-CSF inhalation were determined by the paired *t*-test. The relationship between variations of DLCO-SB and variations of other parameters were also assessed by Pearson**'s** correlation analysis. Data were analyzed using SPSS18.0 statistical software, with significance defined as *P* < 0.05 (two-sided).

## 3. Results

### 3.1. Baseline Characteristics of PAP Patients

Clinical and biochemical characteristics of the PAP patients are listed in Table 1. The mean age at diagnosis was 46.61 ± 11.08 years. Predominantly male patients had PAP (63.11%). Most patients (59.02%) were smokers before diagnosis. Mean serum CEA, CYFRA21-1, and NSE levels were 4.68 ± 0.147 ng/ml, 10.18 ± 2.23 ng/ml, and 201.16 ± 5.69 ng/ml, respectively. Mean DLCO-SB was 63.72 ± 15.82%.

### 3.2. The Relationships between DLCO-SB with Other Parameters in Patients of PAP at Baseline

Table 2 showed that DLCO-SB was negatively correlated with TC, LDL-C, WBCC, neutrophil count, CEA, CYFRA21-1, NSE, and LDH while positively correlated with HDL-C (*P* < 0.05) in patients of PAP at baseline. Nevertheless, we did not observe the statistically significant relationship between DLCO-SB and age, SBP, DBP, TG, apoA1, apoB, IgA, IgG, IgM, IgE, C3, C4, lymphocyte count, and monocyte count.

### 3.3. Changes in Serum DLCO-SB and Other Parameters Six Months after GM-CSF Inhalation Therapy in PAP Patients

Inhaled GM-CSF was administered to 14 patients. HDL-C and DLCO-SB significantly increased while CEA, CYFRA21-1, NSE, and LDH levels significantly decreased six months after GM-CSF inhalation therapy. There were no differences of TC, TG, LDL-C, apoA1, apoB, WBCC, neutrophil count, lymphocyte count, and monocyte count before and after treatment (Table 3).

### 3.4. The Relationship between Variations of DLCO-SB and Variations of Other Parameters after GM-CSF Inhalation Therapy in PAP Patients

**Figure 1:**
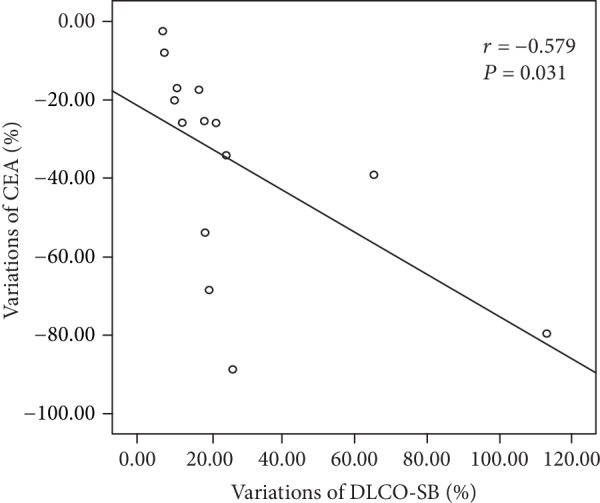
The relationship between variations of DLCO-SB and variations of CEA after inhalation of the GM-CSF treatment in PAP patients.

**Figure 2:**
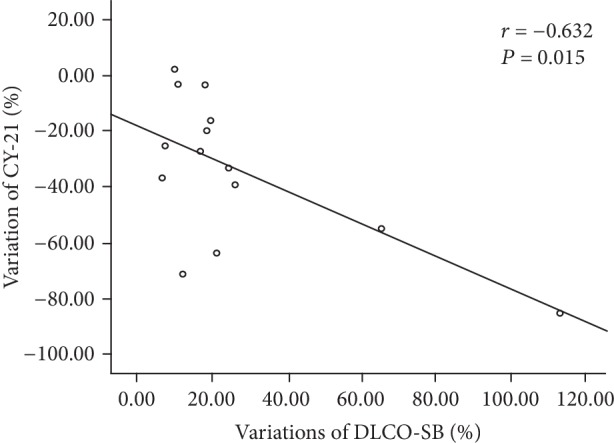
The relationship between variations of DLCO-SB and variations of CYFRA21-1 after inhalation of the GM-CSF treatment in PAP patients.

The increased DLCO-SB was significantly correlated with decreased CEA (*r* = ‐0.579, *P* = 0.031) and CYFRA 21-1 (*r* = ‐0.632, *P* = 0.015) but not with variations of HDL-C, NSE, and LDH (Table 4, Figures 1 and 2).

### 3.5. Comparison of DLCO-SB and Other Parameters between Baseline and Six-Month Follow-Up in PAP Patients without GM-CSF Inhalation Therapy

10 PAP patients without GM-CSF inhalation therapy were enrolled in our study. After six months of follow-up, HDL-C and DLCO-SB significantly decreased while NSE and LDH levels significantly increased in 10 PAP patients without GM-CSF inhalation therapy. There were no differences of TC, TG, LDL-C, apoA1, apoB, WBCC, neutrophil count, lymphocyte count, monocyte count, CEA, and CYFRA21-1 between baseline and 6-month follow-up (Table 5).

### 3.6. The Relationship between Variations of DLCO-SB and Variations of Other Parameters in PAP Patients without GM-CSF Inhalation Therapy after Six Months of Follow-Up

**Figure 3:**
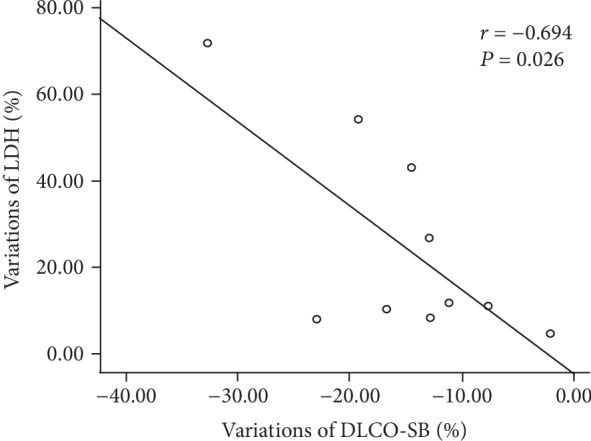
The relationship between variations of DLCO-SB and variations of LDH in PAP patients without inhalation of the GM-CSF treatment after six months of follow-up.

The decreased DLCO-SB was significantly correlated with increased LDH (*r* = ‐0.694, *P* = 0.026) but not with variations of HDL-C, CEA, CYFRA21-1, and NSE (Table 6, Figure 3).

## 4. Discussion

Compared with other similar studies, this retrospective study evaluated valuable serum makers in larger PAP samples. In this study, we measured the levels of various clinical variables in the serum of 122 patients with PAP. Levels of LDH, CEA, NSE, neutrophil count, TC, HDL-C, and LDL-C were significantly distinguished in PAP patients compared with normal subjects. By Pearson's correlation analysis, there was a significant correlation between the level of LDH, CEA, NSE, CYF21-1, TC, HDL-C, and LDL-C in serum and pulmonary dysfunction in PAP patients. In addition, we found that there was a significant inverse correlation between the level of CEA and CYF21-1 and pulmonary function in 14 PAP patients who had been administered inhaled GM-CSF therapy for six months. In 10 PAP patients without GM-CSF therapy, this study showed that an elevation of LDH was observed and a decrease in DLCO after six months of follow-up.

Elevation of serum markers in PAP patients is associated with impaired metabolism through the dysfunction of alveolar macrophages [6, 7]. Although tumor markers were widely used in detecting malignancies, they were also detected in nonmalignant diseases. In recent years, there were researches studying tumor markers such as CEA, CYF21-1, and NSE that might reflect the severity of PAP [8–10]. This study showed that LDH, CEA, and CYFRA 21-1 could be sensitive and useful serum markers for the evaluation of disease activity of PAP. An elevation of LDH can be found in a variety of systemic inflammatory diseases. Early studies showed that the serum level of LDH is often slightly elevated and maybe a marker of the severity of PAP [11]. CEA is a cell surface glycoprotein, whose concentration is high in foetal tissues and a variety of tumors, most commonly those of endodermal origin [12]. In fact, CEA localization has been reported in the lungs of PAP patients [13]. A previous study has reported that in nonmalignant pulmonary diseases, elevation of serum CYFRA 21-1 is due to epithelial damage and its increased production in the epithelium [9].

PAP is a rare disease characterized by the alveolar accumulation of surfactant components, which impairs gas exchange, resulting in respiratory failure. The clinical course is unpredictable. Spontaneous improvement or even cure can occur, and the 5-year actuarial survival is 95% [14]. The most frequent complications are infectious etiology. Whole lung lavage (WLL) has been the first-line therapy since the 1960s [15]. However, WLL is not standardized and international consensus documents are lacking. Moreover, WLL is associated with adverse effects such as infections, fever, convulsions, pneumothorax, pleural effusion, hypoxemia, or even death. 10% of patients with PAP need repeated whole lung lavage [16]. In recent years, new treatments are available such as subcutaneous or inhaled GM-CSF supplementation, plasmapheresis, or rituximab infusions [17]. Inhaled GM-CSF therapy is currently widely used, but the therapy has a slow onset and has limited efficacy in patients with severe pulmonary function decline in the advanced stage. One retrospective study by Soyez et al., which included 13 PAP patients, reported rituximab could not be as a second-line therapy for patients with PAP [18]. Nowadays, oral statin therapy can be a novel pharmacotherapy of PAP. In a study by McCarthy et al., the expression of cholesterol in alveolar is higher in PAP patients. Statin therapy reduces cholesterol accumulation and ameliorates PAP [19]. However, oral statin therapy has not yet been administered in larger PAP population samples. The present study revealed that there was a significant inverse correlation between the level of LDH, CEA, CYF21-1, and DLCO. In the future, we could monitor the disease activity and effectiveness of various therapies for PAP by detecting the level of LDH, CEA, and CYF21-1.

There are some limitations to this study. First of all, the study is a retrospective observational study. Secondly, the mechanisms responsible for PAP have not been investigated in this study. Finally, it remains unresolved from our investigation, which is the best serum marker of PAP.

## 5. Conclusion

Above all, serum LDH, CEA, and CYF21-1 are valuable and sensitive serum makers for the evaluation of disease activity of PAP and may reflect the response to various treatment of PAP.

## Figures and Tables

**Table 1:** The clinical and biochemical properties of 122 PAP patients.

	PAP (*n* = 122)
Age (years)	46.61 ± 11.08
Male/female	77/45
Smoking (yes/no)	72/50
SBP (mmHg)	126.28 ± 10.94
DBP (mmHg)	76.69 ± 7.72
HDL-C (mmol/l)	1.07 ± 0.39
LDL-C (mmol/l)	2.78 ± 0.79
TC (mmol/l)	4.83 ± 0.92
TG (mmol/l)	1.65 ± 0.94
apoA1 (g/l)	1.97 ± 0.79
apoB (g/l)	0.93 ± 0.25
IgA	1.97 ± 0.79
IgG	11.46 ± 2.58
IgM	1.51 ± 0.72
IgE	94.94 ± 11.54
C3	1.05 ± 0.26
C4	0.24 ± 0.08
WBCC (10^9^/l)	6.45 ± 1.56
Neutrophil count (10^9^/l)	3.88 ± 1.14
Lymphocyte count (10^9^/l)	1.89 ± 0.60
Monocyte count (10^9^/l)	0.47 ± 0.12
CEA (ng/ml)	4.68 ± 0.147
CYFRA21-1 (ng/ml)	10.18 ± 2.23
NSE (ng/ml)	20.16 ± 5.69
LDH (IU/l)	284.52 ± 21.95
DLCO-SB (%)	63.72 ± 15.82

Abbreviations: SBP: systolic blood pressure; DBP: diastolic blood pressure; TC: total cholesterol; TG: triacylglyceride; LDL-C: high-density lipoprotein cholesterol; HDL-C: high-density lipoprotein cholesterol; WBCC: while blood cell count; CEA: carcinoembryonic antigen; NSE: neuron-specific enolase; LDH: lactic dehydrogenase; DLCO: diffusion capacity for carbon monoxide.

**Table 2:** Pearson's correlation analysis of DLCO-SB with other parameters in patients of PAP at baseline.

	*r*	*P*
Age (years)	0.116	0.203
SBP (mmHg)	0.073	0.312
DBP (mmHg)	0.086	0.254
HDL-C (mmol/l)	0.467	<0.001
LDL-C (mmol/l)	-0.363	<0.001
TC (mmol/l)	-0.364	<0.001
TG (mmol/l)	0.137	0.135
apoA1 (g/l)	0.175	0.056
apoB (g/l)	0.091	0.320
IgA	-0.112	0.221
IgG	-0.176	0.053
IgM	-0.151	0.098
IgE	0.041	0.658
C3	-0.011	0.905
C4	-0.090	0.325
WBCC (10^9^/l)	-0.278	0.002
Neutrophil count (10^9^/l)	-0.336	<0.001
Lymphocyte count (10^9^/l)	0.075	0.416
Monocyte count (10^9^/l)	0.048	0.591
CEA	-0.440	<0.001
CYFRA21-1	-0.468	<0.001
NSE	-0.416	<0.001
LDH	-0.472	<0.001

**Table 3:** Comparison of DLCO-SB and other parameters before and after inhalation of the GM-CSF treatment in PAP patients.

	Before GM-CSF treatment (*n* = 14)	After GM-CSF treatment (*n* = 14)
HDL-C (mmol/l)	0.86 ± 0.23	1.13 ± 0.32^a^
LDL-C (mmol/l)	2.69 ± 0.64	2.57 ± 0.83
TC (mmol/l)	4.47 ± 0.80	4.38 ± 0.81
TG (mmol/l)	1.58 ± 0.68	1.73 ± 0.93
apoA1 (g/l)	1.03 ± 0.29	1.08 ± 0.30
apoB (g/l)	0.93 ± 0.21	0.89 ± 0.25
WBCC (10^9^/l)	5.12 ± 1.41	5.63 ± 1.91
Neutrophil count (10^9^/l)	2.79 ± 1.14	3.17 ± 1.21
Lymphocyte count (10^9^/l)	1.78 ± 0.30	1.95 ± 0.58
Monocyte count (10^9^/l)	0.38 ± 0.16	0.39 ± 0.11
CEA	4.28 ± 1.28	2.18 ± 1.16^a^
CYFRA21-1	10.97 ± 2.95	6.92 ± 1.55^a^
NSE	21.71 ± 5.01	17.66 ± 3.83^a^
LDH	300.64 ± 15.81	235.00 ± 10.78^a^
DLCO-SB	58.06 ± 10.36	71.01 ± 12.92^a^

Compared with before GM-CSF treatment, ^a^*P* < 0.05.

**Table 4:** The relationship between variations of DLCO-SB and variations of other parameters after inhalation of the GM-CSF treatment in PAP patients.

	*r*	*P*
Variations of HDL-C (%)	-0.283	0.327
Variations of CEA (%)	-0.579	0.031
Variations of CYFRA21-1 (%)	-0.632	0.015
Variations of NSE (%)	-0.429	0.126
Variations of LDH (%)	-0.344	0.228

**Table 5:** Comparison of DLCO-SB and other parameters between baseline and six-month follow-up in PAP patients without inhalation of the GM-CSF treatment.

	Baseline (*n* = 10)	Six-month follow-up (*n* = 10)
HDL-C (mmol/l)	1.33 ± 0.30	1.05 ± 0.31^a^
LDL-C (mmol/l)	2.52 ± 0.56	2.89 ± 0.77
TC (mmol/l)	4.49 ± 0.95	4.91 ± 1.07
TG (mmol/l)	1.76 ± 0.90	1.25 ± 0.75
apoA1 (g/l)	1.18 ± 0.35	1.21 ± 0.22
apoB (g/l)	0.88 ± 0.21	0.99 ± 0.28
WBCC (10^9^/l)	5.47 ± 1.59	5.19 ± 1.31
Neutrophil count (10^9^/l)	3.11 ± 1.37	2.88 ± 1.05
Lymphocyte count (10^9^/l)	1.68 ± 0.41	1.79 ± 0.39
Monocyte count (10^9^/l)	0.31 ± 0.11	0.32 ± 0.08
CEA	2.12 ± 1.85	2.95 ± 1.54
CYFRA21-1	5.42 ± 2.02	7.25 ± 2.42
NSE	15.43 ± 4.41	17.79 ± 5.17^a^
LDH	205.50 ± 23.97	257.30 ± 26.48^a^
DLCO-SB	78.06 ± 15.00	66.81 ± 17.80^a^

Compared with baseline, ^a^*P* < 0.05.

**Table 6:** The relationship between variations of DLCO-SB and variations of other parameters in PAP patients without inhalation of the GM-CSF treatment after six months of follow-up.

	*r*	*P*
Variations of HDL-C (%)	-0.189	0.601
Variations of CEA (%)	-0.483	0.158
Variations of CY-21 (%)	-0.446	0.196
Variations of NSE (%)	-0.545	0.104
Variations of LDH (%)	-0.694	0.026

## Data Availability

Data can be submitted by corresponding authors in case of a request.
